# Muscle architectural and performance adaptations following an 8-week sprint-focused training intervention

**DOI:** 10.1007/s00421-026-06414-3

**Published:** 2026-08-27

**Authors:** Luke R. Stutter, Steven Duhig, David L. Carey, Matthew W. Driller, Charlie J. Davids, Lachlan P. James

**Affiliations:** 1 School of Allied Health, Human Services, & Sport, La Trobe University, Plenty Road and Kingsbury Drive, Bundoora, Melbourne, VIC 3086 Australia; 2 School of Allied Health, Sport and Social Work, Griffith University, Gold Coast, QLD Australia

**Keywords:** Sprinting, Training intervention, Muscle architecture, Acceleration, Maximal velocity, Muscle fascicles

## Abstract

Muscle fascicle length is a key determinant of sprint performance, with lengthening adaptations observed following eccentric strength training or combined sprint and resistance interventions. However, whether sprint-focused training can induce meaningful architectural adaptations, independent of concurrent sport-related training, remains unclear. Fourteen participants (10 female, 4 male; age 21.6 ± 3.3 years; body mass 68.0 ± 11.8 kg) completed an 8-week speed training intervention using a single-group design. Training sessions consisted of maximal velocity and resisted sprinting, technical acceleration drills, and a low volume of back squats to maintain baseline strength, with no additional lower-body training permitted. Vastus lateralis (VL) and biceps femoris long head (BFlh) fascicle length was estimated using 2-D ultrasound, alongside 40-metre sprint, jump and maximal strength tests. Significant increases in fascicle length were observed in the BFlh (left: 2.23 cm, 95%CI 1.75 to 2.70, *p* < 0.0001; right: 2.44 cm, 95%CI 1.99 to 2.90, *p* < 0.0001) and VL (left: 0.32 cm, 95%CI 0.83 to 1.81, *p* < 0.0001; right: 1.48 cm, 95%CI 0.95 to 2.01, *p* < 0.0001). Large effect sizes were evident across all muscles (*g* = 1.40–2.03, 95% CI 0.86 to 2.46). No significant main effects for time were observed for sprint, jump or strength outcomes, however, small effect size improvements were evident for flying 10-m (*g* = -0.27, 95% CI -0.46 to -0.09) performance. These findings indicate that sprint-focused training may be sufficient to elicit meaningful increases in muscle fascicle length, despite small performance change.

**Supplementary Information:**

The online version contains supplementary material available at https://doi.org/10.1007/s00421-026-06414-3.

## Introduction

Sprint running is a critical determinant of performance across numerous sports, underpinning an athlete’s ability to outrun opponents, evade defensive pressure, and exploit open space (Healy et al. 2022; Slawinski et al. 2017). Performance during sprinting is largely governed by the capacity to generate and apply force effectively, with distinct mechanical demands across different phases of the sprint. Faster sprint performance has been associated with the production of large horizontal forces during acceleration, high vertical forces at maximal velocity, and reduced braking forces throughout ground contact (Morin et al. 2011). Central to these mechanical capabilities is the athlete’s ability to generate force rapidly, enabling efficient force application within the brief ground contact times characteristic of high-speed running (Nagahara et al. 2018). These force production characteristics are underpinned by the structural properties of the muscle-tendon unit, particularly muscle architecture. In this context, longer muscle fascicle lengths have been shown to facilitate greater shortening velocities, attributed to a greater number of sarcomeres arranged in series enabling higher absolute shortening rates at a given relative strain (Wickiewicz et al. 1984), during sprinting. Accordingly, variation in muscle architecture represents a key physiological factor influencing sprint performance. Developing a clearer understanding of these relationships is essential for informing targeted training strategies aimed at optimising high-velocity force production and, ultimately, sprint performance.

Physical adaptations in the muscle architecture may occur alongside performance changes in response to training stimuli. Muscle architecture, particularly muscle fibre length and cross-sectional area, play a substantial role in torque-velocity characteristics (Wickiewicz et al. 1984) developed through sport-specific training exposure. Sprinters, who are repeatedly exposed to high shortening velocities and large eccentric loads, possess longer fascicle lengths and smaller pennation angles than non-sprinters (Abe et al. 2000). Shorter fascicles with larger pennation angles are generally associated with greater force-producing capacity, while longer fascicles with smaller pennation angles facilitate faster contraction velocities (Bellinger et al. 2021). An increased number of sarcomeres arranged in series is proposed to enable higher shortening velocities (Abe et al. 2000), which occur in lengthened fascicles following exposure to large eccentric muscle demands (Timmins et al. 2024). Accordingly, faster sprinters typically exhibit greater muscle thickness, smaller pennation angles, and longer fascicles (Kumagai et al. 2000), in addition to larger muscle volumes (Miller et al. 2020; Nuell et al. 2021), compared to slower sprint athletes. Despite this, sprinting is multifactorial and is influenced by neural adaptations, technique, musculoskeletal properties, coordination and force application strategies (Nagahara et al. 2014; Whitacre et al. 2024). Nevertheless, these findings support muscle architectural changes as an important mechanism in enhancing sprint performance. However, it should be noted that much of this evidence is cross-sectional and observational in nature, and the causal role of fascicle length in determining sprint performance remains to be established through longitudinal training research.

Adaptations in pennation angle, muscle thickness and fascicle length vary according to muscle contraction type and velocity during the training exposure. Duhig and colleagues (Duhig et al. 2019) reported that five weeks of eccentric hamstring training resulted in increased muscle fascicle length, whilst concentric training resulted in shorter fascicles across thirty recreationally-active males. Smaller pennation angles were reported in the eccentric group, compared to larger pennation angles in the concentric group, with no change observed in muscle thickness (Duhig et al. 2019). Fascicle lengthening has also been reported across several intervention types; eccentric resistance training, including the Nordic hamstring exercise (Mendiguchia et al. 2020; Timmins et al. 2024); repeat sprint conditioning without concurrent sport training (Thurlow et al. 2025); and mixed interventions incorporating concurrent sprint and sport training (Freeman et al. 2023; Mendiguchia et al. 2020), sprint and resistance training (Blazevich et al. 2003) and combined lower-limb and sprint or Nordic hamstring training interventions (Ripley et al. 2023). Collectively, this evidence suggests that both eccentric and high-velocity training can elicit favourable architectural adaptations in the muscle that may enhance muscle shortening velocities associated with sprint performance.

Despite extensive evidence indicating lengthening of muscle fascicles occurs following sprinting exposure (Blazevich et al. 2003; Freeman et al. 2023; Mendiguchia et al. 2020), each of the studies in question allowed for concurrent sport or resistance training. It is therefore difficult to isolate the specific effects of the intervention from the addition of other training modalities. In the studies which included concurrent sport training, small-moderate effect sizes were observed for sprint measures (Mendiguchia et al. 2020), whilst only positive trends in sprint performance could be ascertained in another (Freeman et al. 2023). Consequently, limited research has directly linked training-induced muscle architectural adaptations to changes in sprint performance following a controlled sprint-focused intervention. Only one study has examined isolated sprint and jump training, which was performed in individuals with cerebral palsy (Gallinger et al. 2025). Although fascicle length increases were observed in the medial gastrocnemius following high-velocity training, the findings cannot be compared to healthy populations.

Increases in fascicle length can be seen as a positive physiological adaptation to sprint running exposure, but the strength of the relationship between muscle architectural adaptations and sprint running exposure is currently unclear. Previous studies involving concurrent training suggest increases in BFlh fascicle lengths are associated with sprint performance improvements of small-moderate effect (Freeman et al. 2023; Mendiguchia et al. 2020). Therefore, the primary aim of this study was to examine whether similar findings would be observed following a sprint-focused training stimulus on muscle architecture and subsequent sprint performance. It was hypothesised that 8-weeks of sprint-focused training would result in increased muscle fascicle lengths in the VL and BFlh muscles, and that these changes would be accompanied by improvements in sprint and jump performance.

## Methods

### Design

**Fig. 1:**
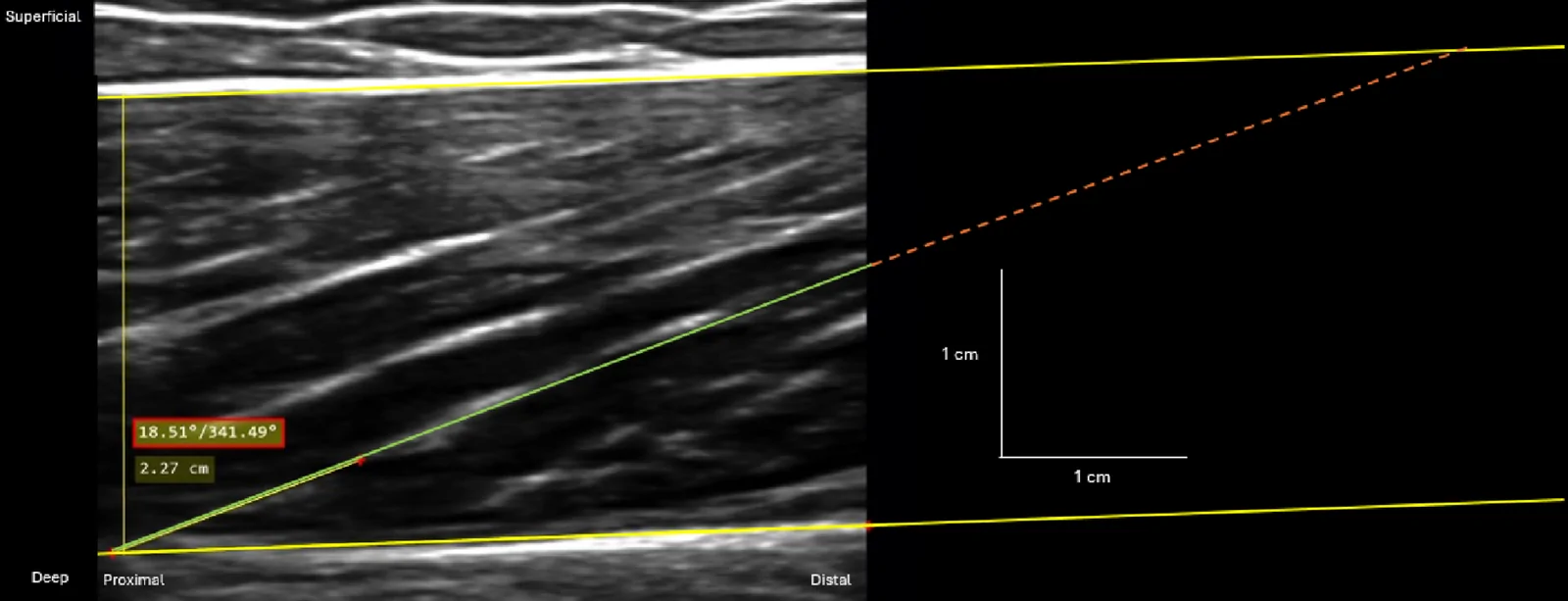
Muscle ultrasound image exemplar including fascicle (pennation) angle, muscle thickness, and manual linear extrapolation of the fascicle in the vastus lateralis

A single-group, repeated measures study design observed the effects of a controlled 8-week sprint-focused training program on muscle architecture and physical performance variables including sprint, jump and strength measures. Participants completed three sessions per week across two four-week training blocks, which were separated by three testing weeks of baseline, mid-test and post-test (Fig. 1). Testing was conducted in one 60-minute session, with no additional training occurring for that week. A sample-size was calculated using G*Power for a medium effect size (f^2^ = 0.15), based on Cohen’s conventions (Cohen 1988) and consistent with previous literature for sprint performance reporting small-to-moderate associations (Blazevich et al. 2003; Freeman et al. 2023; Mendiguchia et al. 2020). Alpha was set at 0.05 and power at 0.80 which estimated 16 participants.

### Participants

Fourteen recreationally-active individuals (10 female, 4 male; age 21.6 ± 3.3 years; body mass 68.0 ± 11.8 kg; training experience 3.9 ± 1.8 years) competing in field sports and classified as tier-two (McKay et al. 2022) participated in the sprint training intervention. Participants needed to be free of any injury and illness and able to perform a maximal back squat exercise with proficient technique, determined by a qualified strength and conditioning coach. The original recruitment pool included 17 participants, however, three withdrew prior to mid-testing either due to injury or personal reasons and were not included in data analysis. Of the 14 remaining participants, one withdrew for a personal reason prior to post-testing, with training adherence averaging 99% of sessions. Both male and female participants were combined in the analysis, similar to previous research (Blazevich et al. 2003). Sex differences have been shown to be less relevant in strength-matched individuals, or when normalised to strength or body mass (Comfort et al. 2024; Nimphius et al. 2019). Ethical approval was granted from the University Human Ethics Committee (HEC24204).

### Procedure

Testing occurred at pre, mid and post-intervention and included 40-metre sprint, countermovement jump (CMJ), drop jump, isometric squat and three repetition maximum (3RM) back squat, as well as ultrasound imaging of the upper thigh. Training sessions were completed three times per week for 8-weeks and emphasised maximal speed, resisted sprint and unresisted acceleration (see supplementary content). In addition, three sets of four repetitions of 80% one-repetition maximum (1RM) back squats, calculated from baseline testing, were performed once per week. This low dose of heavy resistance training was implemented to prevent detraining occurring and not stimulate improvements in heavy dynamic strength, as per recommendations from prior research following ten weeks of ballistic jump training (Cormie et al. 2010b). No other lower body strength training, sprint running or sport training was permitted for the duration of the study.

### Ultrasound

Imagery of the vastus lateralis (VL) and biceps femoris long head (BFlh) muscles in both legs were recorded via a 2-D ultrasound sonography machine (Terason t3200, Terason, Burlington, MA). All images were collected by a single trained assessor. Imaging locations were midpoint of greater trochanter and lateral femoral condyle for VL, and midpoint between ischial tuberosity and lateral tibial condyle for BFlh. Muscle thickness, fascicle (pennation) angle and fascicle length were reported and used to assess morphological adaption in the lower limb, with the average of three images used per muscle. Previous protocols (Duhig et al. 2019; Mairet et al. 2006) using ultrasound sonography were implemented to measure muscle thickness (MT) and fascicle angle (*P*_*sinθ*_), and subsequently estimate muscle fascicle length, in both VL and BFlh muscles of the left and right limbs. Manual linear extrapolation of fascicle lengths was derived from all images across all muscles following previous protocols (Franchi et al. 2020; Potier et al. 2009). Between-session reliability for all muscle architecture measures was established prior to the intervention. Relative reliability was excellent across all metrics; intraclass correlation coefficients (ICC) ranged from 0.91 to 0.95 (95%CI 0.72 to 0.99) for muscle thickness, and from 0.99 to 0.996 (95%CI 0.97 to 0.999) for determining fascicle angles. Absolute reliability and measurement error thresholds demonstrated tight consistency, with coefficients of variation (CV) ranging from 2.39% to 3.73% for muscle thickness (MDC95 0.15 to 0.24 cm) and from 5.89% to 8.53% for fascicle angles (MDC95 2.41 to 3.43 degrees).

### Sprint assessment

Two maximal sprint efforts were performed over 40-metres on an outdoor synthetic grass surface from a standing two-point start, with three minutes rest between. Participants were instructed to wear consistent footwear with a flat sole across testing sessions and ran perpendicular to any ambient wind to control for environmental conditions across testing sessions. The 1080 Motion Sprint device (1080 Motion, Stockholm, Sweden) was used to assess sprint performance. This device is a valid and reliable tool to assess linear sprint performance (Rakovic et al. 2022). The fastest sprint effort was used for analysis and included 5-metre split times up to 40-metres, maximal velocity and maximal acceleration metrics.

### Strength assessment

Jump testing was performed on FP-lite force plates using Forcedecks software (VALD, Brisbane, Australia). Participants performed the drop jump from a 45-centimetre box (Möck et al. 2021) and were cued to jump as high as possible, with minimal contact time on the ground. Only jumps with contact times less than 250 milliseconds were deemed acceptable, and the jump with the greatest reactive strength index (RSI) score was used for analysis (Jarvis et al. 2022). CMJ testing included a maximal jump effort immediately following an eccentric lowering to a self-selected depth. The repetition with the greatest jump height (impulse-momentum method) was used for analysis. Hands were placed on hips to remove arm involvement during both the drop jump and CMJ.

Isometric squat testing was performed using FP-4000 force plates in a VALD isometric frame (VALD, Brisbane, Australia) and involved warm up efforts of approximately 50%, 70% and 90% prior to two maximal efforts. The bar sat across the upper trapezius of the participant with an upright torso and knee angle of 135–140 degrees (Brady et al. 2018). Approximately 100 N of pretension was held for three seconds prior to a maximal push in the squat movement pattern. Peak force needed to be within 250 newtons, or a third attempt was carried out. The test effort with the highest net force at 100 milliseconds (F_100_) was used for analysis.

The 3RM back squat was used to measure maximal dynamic strength. Three sets of eight, five and three repetitions at 50%, 70% and 85% of predicted testing weight were used as warm up. Participants then performed three repetitions to a depth of thigh parallel to the ground, determined by the lead investigator. If the subject successfully performed three repetitions, they were instructed to increase the weight by ≥ 2.5 kg with a rest period of five minutes between testing sets. The process was repeated until the participant could no longer perform three successful repetitions to desired depth. The maximum weight lifted was used for analysis.

### Training program

Participants completed three training sessions lasting 45–60 min each, per week across 8-weeks, separated by mid-testing. Different sprint stimuli (i.e. maximal sprint, resisted sprint, etc.) were used each session in an integrated sprint-focused training program with minimal resistance training including a consistent maintenance volume of back squats once per week. The 1080 Motion Sprint device was used for each session to provide feedback to participants, as well as relative bodyweight resistance for the acceleration training. All sessions were supervised by the lead researcher. Training volume and intensity were progressed throughout the intervention, and a taper was implemented prior to final testing. An example of the training program can be seen in Table 1.

**Table 1:** Example sprint program completed in week 1 of the training intervention

Block 1	Day 1: Max velocity	Day 2: Technical	Day 3: Acceleration
Week 1	Cyclic running: 3 × 20 m Wicket runs: 4 × 20 m (10 m build in) Sprint builds: (2 min rest) 3 × 30 m ~ 80% effort (focus on high knee position) Speed bounds: 3 × 20 m Flying sprints: (2–3 min between) 2 × 10 m fly-ins (20 m build in) 2 × 20 m fly-ins (20 m build in) 1 × 40 m effort	Wall holds: 2 × 4ea Wall reach: 2 × 4ea Wall switch: 2 × 4ea Walking A march: 2 × 4ea A-skip: 2 × 4ea Switch bound: 3 × 3ea Ankle dribbles: 3 × 10 m Accelerations: 4 × 10 m Falling accelerations: 4 × 10 m Accelerations 4 × 20 m Back Squat: 3 × 4 × 80% (~ 4 RIR)	Falling start 4 × 10 m 1080 Sprint: Resisted sprints (2 min between) 20% BW − 3 × 10 m 10% BW − 3 × 10 m 5% BW − 3 × 20 m Unresisted: 3 × 20 m

### Statistical analysis

Descriptive statistics for each outcome variable (mean and standard deviation) are reported at each timepoint (Table 2). Linear mixed models were used to analyse the effects of time on muscle architecture and physical performance outcomes (Douglas et al. 2015). Time (pre-, mid-, post-intervention) was included as a fixed effect, and sex as an interaction term. A random intercept was included for each participant to account for repeated measures.

**Table 2:** Descriptive statistics of participant muscle architecture and performance data with % change from pre-test

Variables	Pre		Mid			Post		
	Mean	SD	Mean	SD	% Change	Mean	SD	% Change
Age (years)	21.6	3.3						
Height (cm)	168.6	9.2						
Weight (kg)	67.4	11.4	67.3	10.9	−0.1	66.1	10.5	−0.1
*Muscle morphology*								
Thickness (cm)								
VL right	2.37	0.29	2.47	0.30	3.9	2.45	0.21	5.4
VL left	2.37	0.26	2.48	0.27	4.9	2.44	0.19	5.4
BFlh right	2.26	0.26	2.42	0.19	8.2	2.45	0.20	10.2
BFlh left	2.35	0.19	2.39	0.21	2.0	2.45	0.17	5.3
*Fascicle pennation angle (θ)*								
VL right	16.81	1.56	15.93	1.21	−5.5	14.94	1.32	−10.6
VL left	16.19	1.64	15.78	1.27	−1.4	15.12	1.17	−4.8
BFlh right	14.81	1.39	14.14	1.07	−4.2	13.42	0.97	−8.7
BFlh left	15.12	1.56	14.27	0.79	−4.8	13.60	0.73	−8.7
*Fascicle length (cm)*								
VL right	8.22	0.79	8.82	0.64	7.3	9.60	1.00	16.8
VL left	8.25	0.71	8.94	0.93	8.3	9.45	1.00	14.5
BFlh right	8.46	0.89	9.71	1.14	14.8	10.79	1.35	27.5
BFlh left	8.37	1.03	9.59	0.91	14.6	10.52	1.02	25.7
*Sprint*								
5 m (s)	1.400	0.084	1.443	0.113	3.1	1.480	0.115	5.2
10 m (s)	2.242	0.128	2.283	0.139	1.8	2.313	0.140	2.5
20 m (s)	3.711	0.224	3.750	0.213	1.1	3.776	0.210	1.1
30 m (s)	5.131	0.330	5.162	0.313	0.6	5.192	0.298	0.5
40 m (s)	6.551	0.452	6.555	0.433	0.1	6.595	0.398	−0.1
10 m Fly (s)	1.420	0.123	1.393	0.127	−1.9	1.403	0.106	−2.2
Maximal velocity (ms^− 1^)	7.198	0.592	7.247	0.642	0.6	7.209	0.548	1.3
Maximal acceleration (ms^− 2^)	6.441	0.614	6.196	0.685	−3.3	5.870	0.658	−7.5
*Strength*								
CMJ jump height (cm)	33.2	5.0	33.4	5.1	1.0	34.1	5.2	4.9
CMJ mean force (N)	1377	313	1404	300	2.2	1360	280	1.7
CMJ peak velocity (ms^− 1^)	2.66	0.19	2.66	0.19	0.4	2.68	0.19	2.2
CMJ contraction time (ms)	666	76	638	62	−3.2	669	74	1.3
DJ contact time (s)	0.193	0.024	0.187	0.017	−2.5	0.192	0.019	0.2
DJ RSI (JH/CT)	1.508	0.285	1.480	0.333	−1.5	1.499	0.298	2.6
ISQ peak force (N)	1582	333	1655	450	3.1	1710	440	7.4
Relative ISQ feak force (N/kg)	24.16	6.81	25.10	7.76	3.3	26.32	7.36	7.6
ISQ force 100ms (N)	485	188	518	152	16.3	528	199	16.4
ISQ force 200ms (N)	920	164	950	162	4.1	985	242	6.5
3RM squat (kg)	84.8	19.7	88.0	19.8	3.9	85.0	18.6	3.5
Relative 3RM squat (kg/kg)	1.26	0.18	1.30	0.18	4.0	1.28	0.18	3.6

*BFlh* biceps femoris long head; *CMJ* countermovement jump; *CT* contact time; *DJ* drop jump; *ISQ* isometric squat; *JH* jump height; *RSI* reactive strength index; *RM* repetition maximum; *SD* standard deviation; *VL* Vastus lateralis

**Fig. 2:**
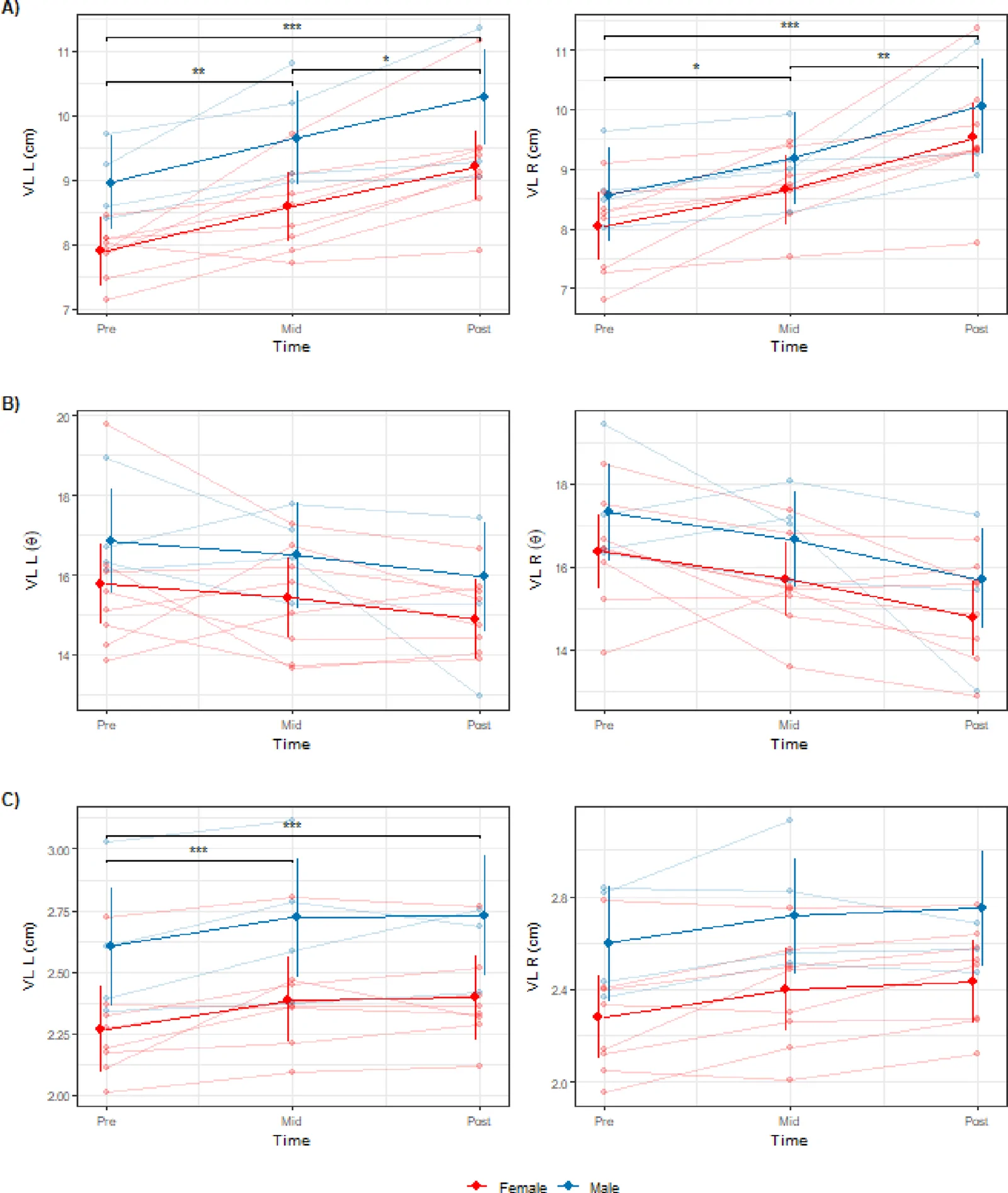
Muscle architecture comparison across time points. **A** Muscle fascicle length (cm); **B** Fascicle pennation angle (degrees); **C** Muscle thickness (cm); *VL* vastus lateralis; *L* left; *R* right. * *p* ≤ 0.05; ** p<0.01; *** *p* ≤ 0.001

**Fig. 3:**
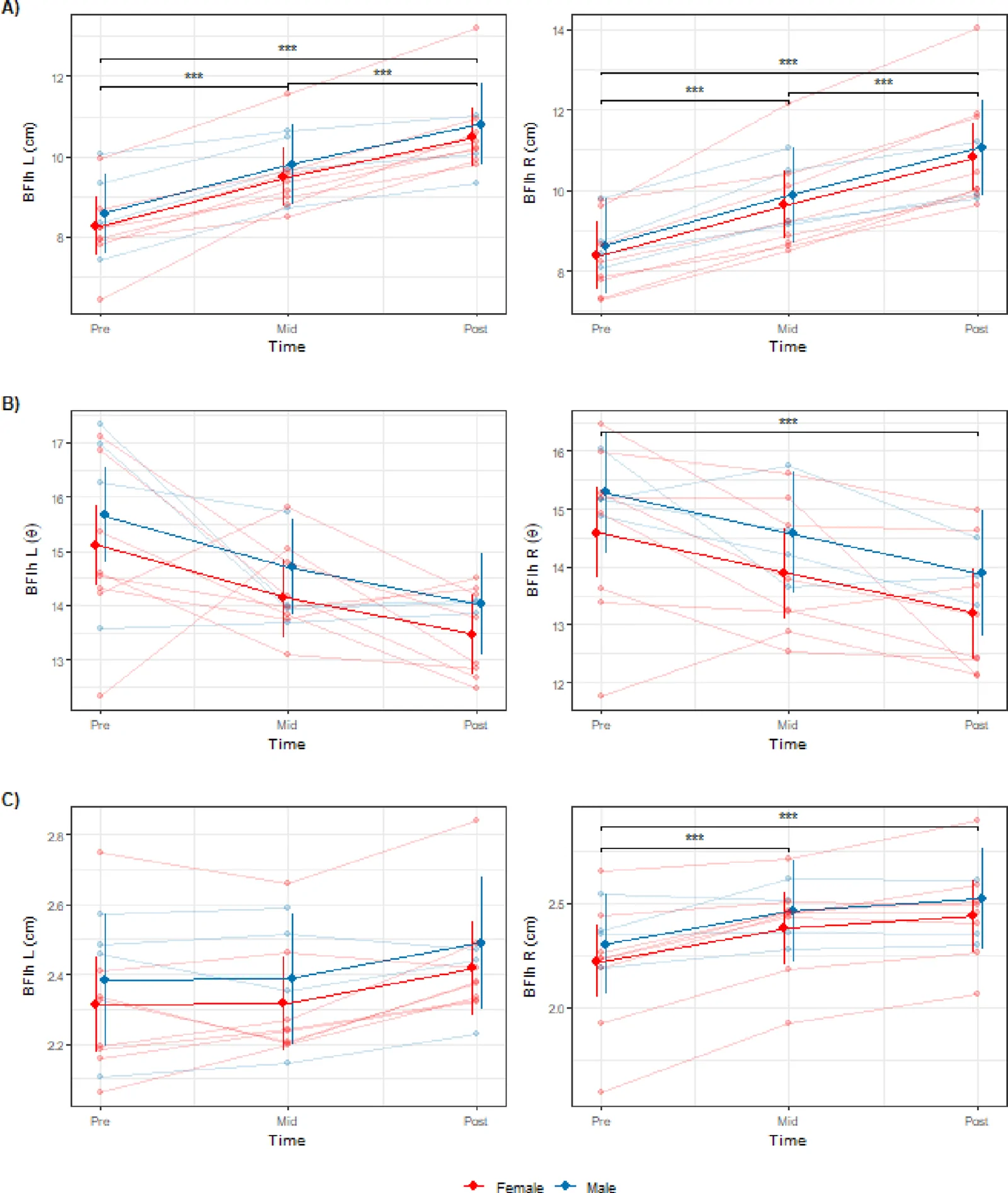
Muscle architecture comparison across time points. **A** Muscle fascicle length (cm); **B** Fascicle pennation angle (degrees); **C** Muscle thickness (cm); *BFlh* biceps femoris long head; *L* left; *R* right; *VL* vastus lateralis. * *p* ≤ 0.05; *** *p* ≤ 0.001

**Fig. 4:**
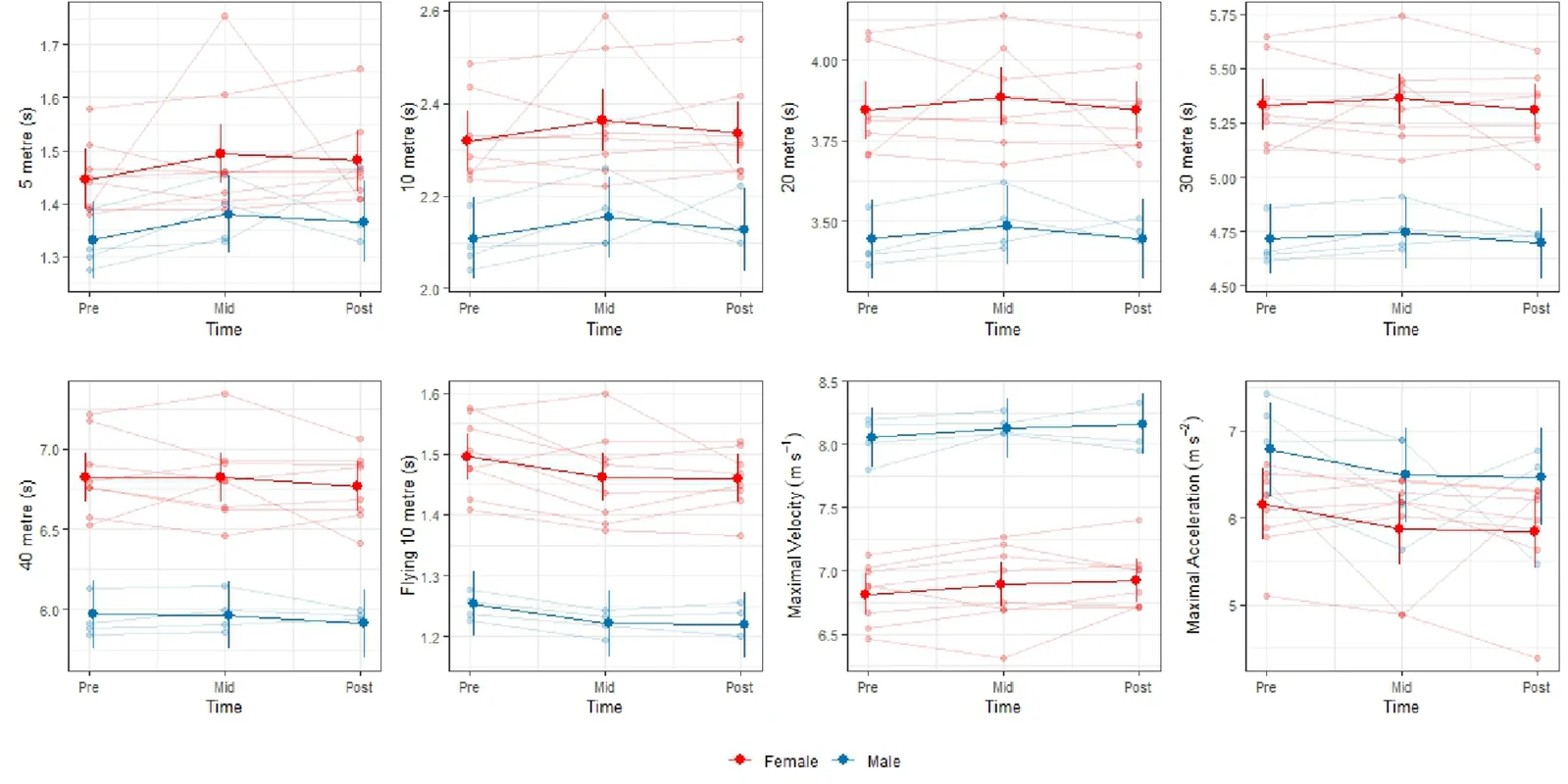
Sprint measure comparison across time for 5–40 m, flying 10-metres, maximal velocity and maximal acceleration

Model fits were visualised for muscle architecture and sprint outcome variables (Figs. 2 , 3 and 4), and all model summary statistics and coefficient estimates are reported in supplementary material (Supplementary Tables 1–5). Statistical significance of main and interaction effects was assessed via a type III analysis of variance (ANOVA) using Satterthwaite’s method (Kuznetsova et al. 2017). Model assumptions (e.g., normality of residuals, homogeneity of variances, multicollinearity) were assessed using the performance package in R (Version 4.3.3). The Bonferroni correction was applied across separate variable families to balance Type I error control with sensitivity. For the muscle architecture family (12 comparisons), the adjusted threshold was set at *p* < 0.0041, and for the performance family (13 comparisons), the adjusted threshold was set at *p* < 0.0038. For significant effects, post-hoc pairwise comparisons were performed using the emmeans package (Lenth 2023).

Muscle architecture and sprint outcome variables at each timepoint are reported, irrespective of statistical significance, as this was the primary outcome comparison for the study. To quantify the magnitude of changes over time, bias-corrected standardized mean differences (Hedges’ *g*) and corresponding 95% confidence intervals were calculated for all pairwise comparisons (Pre–Mid, Mid–Post, and Pre–Post) using the effectsize package in R (version 4.4.3). Effect sizes were computed using the average variance of the compared timepoints (Lakens 2013). Hedges’ *g* was selected over Cohen’s *d* to mathematically correct for small-sample bias, providing more accurate population effect estimates (Hedges 1981). 95% confidence intervals were calculated based on the standard error of Hedges’ *g*. The magnitudes of Hedges’ *g* was interpreted using conventional thresholds (Cohen 1988), whereby values of 0.20–0.49 were considered small, 0.50–0.79 moderate, and ≥ 0.80 large, and are reported alongside percentage change. Spearman’s rank correlation coefficients (ρ) were used to evaluate relationships between individual change scores for selected performance and architectural variables.

## Results

Participant characteristics are presented in Table 2 alongside muscle architecture and performance measures. The final sample size (*n* = 14) was smaller than that determined by the a priori power analysis, due to participant withdrawal.

Linear mixed modelling revealed significant main effects of time for muscle architecture variables in both the BFlh and VL muscles. Post-hoc pairwise comparisons of estimated marginal means at individual time points were significant following Tukey adjustment for BFlh fascicles in the left (mid – pre: 1.22 cm, 95%CI 0.77 to 1.68, *p* < 0.0001; post – pre: 2.23 cm, 95%CI 1.75 to 2.70, *p* < 0.0001; post – mid: 1.00 cm, 95%CI 0.53 to 1.47, *p* = < 0.0001) and right (mid – pre: 1.25 cm, 95%CI 0.81 to 1.70, *p* < 0.0001; post – pre: 2.44 cm, 95%CI 1.99 to 2.90, *p* < 0.0001; post – mid: 1.19 cm, 95%CI 0.73 to 1.65, *p* = < 0.0001). Significant pairwise comparisons were also observed for muscle thickness (mid – pre: 0.16 cm, 95%CI 0.07 to 0.24, *p* < 0.0003; post – pre: 0.22 cm, 95%CI 0.13 to 0.30, *p* < 0.0001) and fascicle angle (post – pre: −1.41 degrees, 95%CI −2.17 to −0.65, *p* = 0.0004) in the right BFlh. For VL, significant pairwise comparisons were revealed for the right (mid – pre: 0.60 cm, 95%CI 0.09 to 1.11, *p* = 0.0190; post – pre: 1.48 cm, 95%CI 0.95 to 2.01, *p* < 0.0001; post – mid: 0.88 cm, 95%CI 0.35 to 1.40, *p* = 0.0011), and left fascicles (mid – pre: 0.69 cm, 95%CI 0.22 to 1.16, *p* = 0.0039; post – pre: 1.32 cm, 95%CI 0.83 to 1.81, *p* < 0.0001; post – mid: 0.63 cm, 95%CI 0.14 to 1.12, *p* = 0.0103), as well as muscle thickness in the left side (mid – pre: 0.12 cm, 95%CI 0.05 to 0.19, *p* = 0.0009; post – pre: 0.13 cm, 95%CI 0.06 to 0.20, *p* = 0.0006). Muscle fascicle changes incurred large effect size increases in length at post-test for both VL and BFlh (14.5–27.5%, *g* = 1.40–2.03, 95% CI 0.86 to 2.46), with moderate-large effect size increases in muscle thickness (5.3–10.2%, *g* = 0.53–0.84, 95% CI 0.21 to 1.16) and reduced fascicle angle (−4.8 – −10.6%, *g* = −1.15 – −0.50, 95% CI −1.90 to 0.12).

There were no significant main effects observed for sprint performance across time for any acceleration or maximal velocity measures. Sprint performance at post-test revealed small effect size performance changes for 5-metre (5.2%, *g* = 0.35, 95% CI 0.02 to 0.69), maximal acceleration (−7.5%, *g* = −0.44, 95% CI −0.85 to −0.03), and flying 10-metre (−2.2%, *g* = −0.27, 95% CI −0.46 to −0.09) variables. All other sprint measures variables were trivial.

Jump and strength outcomes included CMJ, drop jump, isometric squat and 3RM back squat measures. No significant main effects were observed for any jump or strength variable across time. Small effect size changes were observed at post-test for CMJ jump height and peak velocity measures (2.2–4.9%, *g* = 0.29–0.31, 95% CI 0.10 to 0.52), as well as isometric strength measures (7.4–7.6%, *g* = 0.22–0.30, 95% CI −0.03 to 0.60). All other measures were considered trivial.

Sex was included as a covariate in the linear mixed model, with males demonstrating significantly faster sprint times and greater strength and jump measures than females. Significant main effects of sex were observed for maximal velocity (*p* < 0.001), flying 10-metre (*p* < 0.001), 40-metre (*p* = 0.002), 30-metre (*p* = 0.005), CMJ mean force (*p* = 0.006), 20-metre sprint (*p* = 0.013), and 3RM back squat (*p* = 0.047), following Bonferroni correction. No significant main effects of sex were observed for muscle architecture variables, isometric strength, or remaining jump and sprint measures.

Spearman’s rank correlation coefficients (ρ) were used to examine the relationship between training-induced changes in muscle architecture and flying 10-metre sprint performance. Change scores were structured such that negative values denoted an improvement in sprint performance (i.e., decreased sprint time) and positive values denoted increased fascicle length. Although the direction of the relationships indicated that greater increases in fascicle length correlated with greater reductions in sprint times, the findings were trivial to small and failed to reach statistical significance for both average vastus lateralis (ρ = −0.309, *p* = 0.355) and biceps femoris long head fascicle lengths (ρ = −0.145, *p* = 0.670).

## Discussion

The primary aim of this study was to examine the effects of an 8-week sprint-focused training intervention, in the absence of concurrent sport practice, on muscle architecture adaptations and sprint performance measures. Given the single-group design and absence of a control group, the findings should be interpreted as observational in nature, and causation of the observed changes to the training intervention should be made with caution. It was hypothesised that elongation of muscle fascicles and sprint performance improvement would occur following the intervention. Significant main effects of time were observed for muscle architecture, but not for any performance measures. Specifically, muscle fascicle length increased in left and right BFlh and VL. Contrary to the hypothesis, no significant main effects for time were observed for any sprint performance measures, although small effect size changes in some sprint measures were demonstrated. No sex-based differences were detected for training-induced muscle architectural adaptations following Bonferroni correction.

### Muscle architecture

Muscle fascicle length showed significant lengthening following 8-weeks of sprint-focused training in both BFlh and VL muscles. In addition, right BFlh muscle thickness and fascicle angle fig. 3, as well as left VL muscle fig. 2- thickness demonstrated statistically significant differences following the training intervention. Furthermore, moderate to large effect sizes at post-testing were observed, indicating a reduction in fascicle angle, an increase in muscle thickness and subsequent elongation of estimated muscle fascicle lengths for both left and right sides. However, these findings should be interpreted cautiously. Previous investigations have reported strong associations between faster sprint performances and longer fascicle lengths, particularly in the hamstrings (Abe et al. 2000; Duhig et al. 2019; Kumagai et al. 2000). Such adaptations have been hypothesised to arise from substantial eccentric loading during the touchdown and end swing phase of sprinting (Kumagai et al. 2000). However, this mechanistic explanation remains speculative.

While previous investigations demonstrate elongated muscle fascicles following concurrent sprint and sport training (Freeman et al. 2023; Mendiguchia et al. 2020), this was the first study to examine sprint training in the absence of emphasised concurrent strength or sport training, and to detect corresponding changes in muscle architecture. It was hypothesised that muscle fascicles would lengthen as consequence of the high eccentric demands imposed during speed training. The findings are consistent with this hypothesis observing significantly longer estimated muscle fascicles in both BFlh and VL muscles, with moderate to large effect. Importantly, the estimation method assumes linear muscle fascicles and potential measurement variability in the calculations may arise. Existing evidence suggests that either eccentric overload or a pronounced velocity emphasis during muscle contraction is necessary to induce fascicle elongation (Abe et al. 2000). Previous eccentric strength training interventions have reported increases in fascicle length of 1.08–1.40 cm in the BFlh (Duhig et al. 2019; Timmins et al. 2021) and 0.96 cm in the VL (Nimphius et al. 2012). Similarly, studies employing concurrent sprint and sport training studies observed BFlh fascicle lengthening of 1.67–1.95 cm (20–23%) (Freeman et al. 2023) and 1.66 cm (16%) (Mendiguchia et al. 2020) after twice weekly sessions for six weeks. By comparison, the current study induced 1.20–1.38 cm (14.5–16.8%) in the VL and 2.15–2.33 cm (25.7–27.5%) changes in estimated BFlh fascicle length after thrice weekly sessions over 8-weeks.

To the authors’ knowledge, this represents the first investigation to detect adaptations in muscle fascicle architecture following sprint-focused training without concurrent sport participation. Collectively, these findings are consistent with the suggestion that a sprint-focused training program can induce meaningful fascicle elongation, with magnitudes comparable to those reported following eccentric resistance training or concurrent sprint and sport training interventions. It should be acknowledged, however, that the intervention included weekly back squats at 80% 1RM and resisted sprinting, both of which may independently contribute to architectural remodelling, and the relative contribution of each component cannot be fully isolated. The back squat dose was deliberately low and substantially below volumes typically associated with architectural change in the resistance training literature (Cormie et al. 2010b), suggesting sprint exposure remained the dominant training stimulus.

### Sprint performance

Despite the aforementioned changes to muscle fascicle length, sprint performance did not significantly change across the intervention period (Fig. 4). The study identified that acceleration and maximal sprint phases differed in their response, with maximal acceleration and 5-metre sprint performance demonstrating small effect size decrements at post-test, while flying 10-metre sprint time indicated faster sprint performance with small effect size change. One plausible explanation for the divergent acceleration and maximal velocity responses is that variability in early acceleration may have disproportionately influenced cumulative split times, consistent with modelling suggesting the initial 0–10 m exhibits the greatest inter-trial variability in sprint performance (Healy et al. 2022).

However, the absence of statistically significant sprint improvement despite large architectural adaptations warrants deeper consideration. While cross-sectional literature historically assumes a tight, linear coupling between long muscle fascicles and superior sprinting capacity (Abe et al. 2000; Kumagai et al. 2000), our findings challenge this simplistic interpretation. A lack of acute coupling was confirmed at the individual level by our exploratory correlation analyses. Although the direction of these individual relationships indicated that greater increases in fascicle length correlated with greater reductions in flying sprint performance, the correlations were trivial to small and failed to reach statistical significance. This suggests that muscle fascicle length and sprint performance may not be as tightly coupled as often assumed.

It is possible that fascicle length adaptation may precede, rather than immediately produce, improvements in sprint performance. Structural changes to the muscle, including the addition of sarcomeres in series, may require concurrent neuromuscular adaptations, such as increased motor unit recruitment, enhanced firing rates, and improved inter-muscular coordination, before the architectural change can be expressed as a meaningful performance gain (Folland and Williams 2007). These neuromuscular adaptations were not assessed in the present study, representing a gap in the mechanistic interpretation of the findings. Furthermore, sprint performance is inherently multifactorial, depending not only on muscle architecture but also on tendon stiffness, force application technique, coordinative skill, and the capacity to generate and orient ground reaction forces appropriately across acceleration and maximal velocity phases (Morin et al. 2011; Nagahara et al. 2018). It is therefore unlikely that fascicle length change alone, in the absence of concurrent adaptation across these other determinants, would be sufficient to produce measurable sprint improvement. Finally, the 8-week intervention duration may have been insufficient for architectural adaptation to transfer to performance. Evidence from eccentric training interventions suggests that fascicle length changes can occur within four to six weeks, yet sprint performance improvements may require longer exposure to allow neuromuscular and technical factors to consolidate (Thurlow et al. 2025; Timmins et al. 2021). Collectively, these considerations suggest that the null performance findings do not necessarily undermine the functional relevance of the architectural adaptations observed, but rather highlight that structural and performance adaptation may operate on different timescales and require different training emphases to produce concurrent improvements.

### Strength and jump performance

No significant main effects were identified for any performance measures including CMJ, drop jump, isometric squat, or 3RM back squat. Small effect size changes were evident at post-test for CMJ jump height and peak velocity, and isometric squat peak force and relative peak force. Drop jump RSI was anticipated to incur larger improvements due to repeated ground contact times during maximal sprint exposures, however, trivial changes were observed. Although RSI and maximal sprint performance have previously demonstrated strong correlations, the present findings suggest that sprint training may not meaningfully enhance drop jump performance in recreationally active individuals. Despite this, jump performance and maximal strength measures were maintained throughout the intervention. These findings indicate that sprint training incorporating resisted accelerations and maximal velocity exposure, combined with minimal heavy resistance training, is sufficient to maintain maximal dynamic strength for up to 8-weeks. This supports previous research following ballistic jump training over ten weeks (Cormie et al. 2010a, b). The present study is the first to demonstrate that maximal dynamic strength can be preserved during a sprint-focused training program when performed once per week.

### Sex differences

Male participants demonstrated superior sprint performance across 20–40 m splits, flying 10-m sprint times, and maximal velocity measures, consistent with established sex-based differences in sprint performance (Hunter et al. 2023). In contrast, no statistically significant training-induced differences in muscle architecture were detected between sexes, given the unequal and small sub-sample distribution (4 males, 10 females). Therefore, the absence of statistically significant sex interactions must not be interpreted as definitive evidence that males and females respond similarly to the stimulus; rather, it reflects a limited statistical capacity to detect true between-sex differences. Regarding strength, the 3RM back squat differed between sexes, however, this difference was no longer evident when normalised to body mass, as per previous research (Comfort et al. 2024; Nimphius et al. 2019). Overall, while the findings outline absolute differences in performance capacity, sex-specific architectural adaptation patterns cannot be drawn, and future research with larger, balanced cohorts is required to evaluate potential sex-specific remodelling.

### Limitations

The authors acknowledge several limitations to the study. Firstly, the lack of a control group limits the ability to attribute observed changes solely to the sprint training intervention and measurement error or natural variation cannot be excluded. This reduces the internal validity of the findings and limits the strength of causal inferences that can be drawn. While the inclusion of a non-exercising control group would have strengthened the study design, it was not feasible to request an additional group of participants to refrain from exercise for the duration of the 8-week intervention, particularly given ethical and practical considerations. Consequently, the findings should be interpreted with caution, and future research employing controlled or randomized designs is warranted. Secondly, the relatively small sample size limited statistical power and may have increased the risk of a Type II error for performance metrics. Although effect sizes were reported to aid interpretation, and some non-significant findings should be interpreted with caution, the clear divergence between observed architectural adaptations and null sprint performance changes likely reflects a true physiological decoupling rather than a simple artifact of statistical underpowering. Menstrual cycle phase and hormonal contraceptive use were not monitored among female participants, which was not feasible within this field-based intervention. However, recent systematic reviews have found no consistent effect of either factor on resistance training adaptations (Nolan et al. 2024) or athletic performance (Meignié et al. 2021), and future research should incorporate standardised verification methods (Elliott-Sale et al. 2021) to determine whether these factors moderate architectural adaptations specifically. Finally, muscle architecture was assessed via two-dimensional ultrasound which requires estimation of muscle fascicle length beyond the visible field of view. Although this method has been validated (Duhig et al. 2019; Klimstra et al. 2007), it remains susceptible for measurement error as it assumes linear fascicles, and does not account for fascicle curvature. This limitation is particularly relevant for the BFlh, which possesses longer and more curved fascicles than the VL at rest, potentially leading to overestimation of true fascicle length compared to extended field of view methods (Franchi et al. 2020). Future studies should utilise ultrasound probes with an extended field of view to enable direct visualisation of full fascicle length, thereby improving measurement accuracy.

## Conclusion

Muscle fascicle length increased in both the biceps femoris long head and vastus lateralis muscles following 8-weeks of sprint-focused training, with large effect sizes. While overall sprint performance did not demonstrate significant main effects relative to baseline, small effect size changes were observed in maximal velocity-related measures. These findings are consistent with the suggestion that favourable architectural adaptations to sprint exposure may occur in the absence of measurable changes in sprint performance. Practitioners can expect meaningful fascicle lengthening of 15–28% following 8 weeks of thrice-weekly sprint-focused training incorporating maximal velocity, resisted sprinting, and technical acceleration work. Practitioners should not anticipate concurrent sprint performance improvement on these timescales, and longer training durations or additional neuromuscular stimuli may be required before architectural adaptation translates to measurable performance gains. Further research is required to determine whether architectural change potentially precedes detectable performance enhancement.

## Supplementary Information

Below is the link to the electronic supplementary material.

Supplementary Material 1
