# Supplementary material for: Muscle architectural and performance adaptations following an 8-week sprint-focused training intervention
Source: Eur J Appl Physiol. 2026 Aug 27;126(11):6097–110. doi: 10.1007/s00421-026-06414-3 (PMC13751751; doi:10.1007/s00421-026-06414-3)
Supplement: Supplementary file 1 [file 421_2026_6414_MOESM1_ESM.docx]

Supplementary Tables

Table S1: Muscle architecture changes in the Biceps Femoris Long Head (BFlh).

Note: R, right; L, left; T, thickness; A, fascicle pennation angle; F, Fascicle.

Table S2: Muscle architecture changes in the Vastus Lateralis

Note: R, right; L, left; T, thickness; A, fascicle pennation angle; F, Fascicle.

Table S3: Sprint performance measures at pre-, mid- and post-timepoint.

Table S4: Jump performance measures at pre-, mid-, and post-timepoints

Table S5: Strength performance measures at pre-, mid-, and post-timepoints

Table S6: Full sprint program completed over the 8-week speed intervention.

| **Block 1** | **Day 1: Max Velocity** | **Day 2: Technical** | **Day 3: Acceleration** |
| --- | --- | --- | --- |
| **Week 1** | Cyclic Running: 3 x 20m Wicket runs: 4 x 20m (10m build in)  Sprint builds: (2 mins rest) 3x30m ~80% effort (focus on high knee position)  Speed Bounds: 3 x 20m  Flying sprints: (2-3 mins between) 2x10m fly-ins (20m build in) 2x20m fly-ins (20m build in)  1 x 40m effort | Wall Holds: 2 x 4ea Wall Reach: 2 x 4ea Wall Switch: 2 x 4ea Walking A March: 2 x 4ea  A-Skip: 2 x 4ea Switch bound: 3 x 3ea Ankle dribbles: 3 x 10m  Accelerations: 4 x 10m Falling accelerations: 4 x 10m Accelerations 4 x 20m  Back Squat: 3 x 4 x 80% (~4 RIR) | Falling start 4 x 10m  1080 Sprint: Resisted Sprints (2 mins between) 20% BW - 3 x 10m 10% BW - 3 x 10m 5% BW - 3 x 20m  Unresisted: 3 x 20m |
| **Week 2** | Cyclic Running: 3 x 20m Wicket runs: 4 x 20m (10m build in)  Sprint builds: (2 mins rest) 3 x 30m ~80% (focus on high knee position)  Speed Bounds: 3 x 20m  Flying sprints: (2-3 mins between) 2 x 10m fly-ins (20m build in) 2 x 20m fly-ins (20m build in)  1 x 40m effort | Wall Holds: 3 x 4ea Wall Reach: 2 x 4ea Wall Switch: 2 x 4ea Walking A March: 2 x 4ea  A-Skip: 3 x 4ea Switch bound: 3 x 3ea Ankle/calf dribbles: 3 x 10m  Accels/power outs: 4 x 10m Falling accels: 4 x 10m Power outs: 4 x 20m  Back Squat: 3 x 4 x 80% (~4 RIR) | Falling start 4 x 10m  1080 Sprint: Resisted Sprints (2 mins between) 20% BW - 4 x 10m 10% BW - 4 x 10m 5% BW - 4 x 20m  Unresisted: 3 x 20m |
| **Week 3** | Cyclic Running: 2 x 20m Wicket runs: 3 x 20m (10m build in)  Sprint builds: (2 mins rest) 2 x 30m ~80% (focus on high knee position)  Speed Bounds: 3 x 20m  Flying sprints: (2-3 mins between) 3 x 20m fly-ins (20m build in) 3 x 30m fly-ins (20m build in)  1 x 40m effort | Wall Reach: 1 x 4ea Wall Switch: 2 x 4ea Wall Double Switch: 3 x 4ea  A-Skip: 3 x 4ea A-skip Double Switch: 2 x 4  Ankle-calf-knee dribbles: 3 x 20m Mini hurdle bounces (DL): 3 x 10 Switch bound: 3 x 3ea  Accels/power outs: 4 x 10m Falling accels: 4 x 10m Power outs: 4 x 20m  Back Squat: 3 x 4 x 80% (~4 RIR) | SL Hinge to Accel 4 x 10m  1080 Sprint: Resisted Sprints (2 mins between) 20% BW - 6 x 10m 10% BW - 6 x 10m 5% BW - 4 x 20m  Unresisted: 3 x 20m |
| **Week 4** | Cyclic Running: 2 x 20m Wicket runs: 2 x 20m (10m build in)  Sprint builds: (2 mins rest) 2x30m ~80% (focus on high knee position)  Speed Bounds: 3 x 20m  Flying sprints: (2-3 mins between) 3x20m fly-ins (20m build in) 3x30m fly-ins (20m build in)  2 x 40m effort | Wall Reach: 1 x 4ea Wall Switch: 2 x 4ea Wall Double Switch: 2 x 4ea  A-Skip: 1 x 4ea A-skip Double Switch: 2 x 4  Ankle-calf-knee dribbles: 2 x 20m Mini hurdle bounces (DL): 2 x 10 Switch bound: 3 x 3ea  Accels/power outs: 5 x 10m Falling accels: 4 x 10m Power outs: 5 x 20m  Back Squat: 4 x 4 x 80% (~4 RIR) | SL Hinge to accel - 4 x 10m  1080 Sprint: Resisted Sprints (2 mins between) 20% BW - 6 x 10m 10% BW - 6 x 10m 5% BW - 4 x 20m  Unresisted: 2 x 20m 1 x 30m |
| **Block 2** | **Day 1: Max Velocity** | **Day 2: Technical** | **Day 3: Acceleration** |
| **Week 5** | Cyclic Running: 2 x 20m Wicket runs: 3 x 20m (10m build in) Speed Bounds: 3 x 20m  Sprint builds: (2 mins rest) 2x30m ~90% (focus on high knee position)  Flying sprints: (2-3 mins between) 3 x 10m fly-ins (20m build in) 3 x 20m fly-ins (20m build in)  2 x 20m 1 x 40m effort | Wall Switch: 2 x 4ea Wall Doubles 2 x 4ea A skip/Switch: 2 x 4ea A Double Switch: 2 x 4  Projection/Switch bound: 4 x 3ea Accels/power outs: 4 x 10m Banded release: 3 x 10m/10m (3x 20m)  Ankle-calf-knee dribbles: 3 x 20m  Back Squat: 3 x 4 x 80% (~4 RIR) | SL Hinge to accel - 4 x 5m  1080 Sprint: Resisted Sprints (2 mins between) 20% BW - 4 x 10m 15% BW - 4 x 10m 10% BW - 3 x 10m 5% BW - 3 x 20m  Unresisted - 3 x 20m |
| **Week 6** | Cyclic Running: 2 x 20m Wicket runs: 3 x 20m (10m build in) Speed Bounds: 3 x 20m Sprint builds: (2 mins rest) 2x30m ~90% (focus on high knee position)  Flying sprints: (2-3 mins between) 4 x 10m fly-ins (20m build in) 3 x 20m fly-ins (20m build in)  2 x 20m 1 x 40m effort | Wall Switch: 2 x 4ea Wall Doubles 2 x 4ea A skip/Switch: 2 x 4ea A Double Switch: 2 x 4  Projection/Switch bound: 4 x 3ea Accels/power outs: 6 x 10m Banded release: 4 x 10m/10m (3x 20m)  Ankle-calf-knee dribbles: 3 x 20m  Back Squat: 3 x 4 x 80% (~4 RIR) | SL Hinge to accel - 4 x 5m  1080 Sprint: Resisted Sprints (2 mins between) 20% BW - 4 x 10m 15% BW - 4 x 10m 10% BW - 4 x 10m 5% BW - 3 x 10m 5% BW - 3 x 20m  Unresisted - 3 x 20m |
| **Week 7** | Cyclic Running: 2 x 20m Wicket runs: 2 x 20m (10m build in) Speed Bounds: 3 x 20m  Sprint builds: (2 mins rest) 2x30m ~90% (focus on high knee position)  Flying sprints: (2-3 mins between) 4x10m fly-ins (10m build in) 2x20m fly-ins (20m build in)  2 x 20m, 2 x 40m effort | Wall Switch: 2 x 4ea Wall Doubles 2 x 4ea A skip/Switch: 2 x 4ea A Double Switch: 2 x 4  Projection/Switch bound: 4 x 3ea Ankle-calf-knee dribbles: 4 x 20m Accels/power outs: 6 x 10m, 4x 20m  Back Squat: 3 x 4 x 80% (~4 RIR) | SL Hinge to accel - 4 x 5m  1080 Sprint: Resisted Sprints (2 mins between) 20% BW - 2 x 10m 10% BW - 6 x 10m 5% BW - 6 x 10m 5% BW - 3 x 20m  Unresisted - 3 x 20m |
| **Week 8** | Cyclic Running: 2 x 20m Wicket runs: 2 x 20m (10m build in) Speed Bounds: 3 x 20m  Flying sprints: (2-3 mins between) 2x10m fly-ins (10m build in) 2x20m fly-ins (20m build in)  1 x 20m, 1 x 40m effort | Wall Switch: 2 x 4ea Wall Doubles 2 x 4ea A skip/Switch: 2 x 4ea A Double Switch: 2 x 4  Projection/Switch bound: 2 x 3ea Ankle-calf-knee dribbles: 2 x 20m Accels/power outs: 3 x 10m, 3x 20m  Back Squat: 3 x 4 x 80% (~4 RIR) | SL Hinge to accel - 4 x 5m  1080 Sprint: Resisted Sprints (2 mins between) 20% BW - 2 x 10m 10% BW - 4 x 10m 5% BW - 4 x 20m  Unresisted - 2 x 20m |
